# Distal Insertional Footprint of the Brachialis Muscle: 3D Morphometric Study

**DOI:** 10.1155/2015/786508

**Published:** 2015-11-10

**Authors:** Srinath Kamineni, Abdo Bachoura, William Behrens, Ellora Kamineni, Andrew Deane

**Affiliations:** Elbow Shoulder Research Centre, University of Kentucky Department of Orthopaedic Surgery and Sports Medicine, Kentucky Clinic, 740 South Limestone, Lexington, KY 40536-0284, USA

## Abstract

*Objective*. The purpose of this study is to describe the three-dimensional morphometry of the brachialis muscle at its distal attachment to the ulna. *Methods*. Fifty cadaveric elbows were dissected and the brachialis distal insertion was isolated on the ulna bone and probed with a three-dimensional digitizer, to create a three-dimensional model of the footprint. Measurements and analysis of each footprint shape were recorded and compared based on gender and size. *Results*. There was significant gender difference in the surface length (*P*= 0.002) and projected length (*P*= 0.001) of the brachialis footprint. The shapes of the footprint also differed among the specimens. *Conclusion*. The shape of the brachialis muscle insertion differed among all the specimens without significant variation in gender or sides. There was also a significant difference in muscle length between males and females with little difference in the width and surface area. *Significance*. The information obtained from this study is important for kinematic understanding and surgical procedures around the elbow joint as well as the understanding of the natural age related anatomy of the brachialis footprint morphology.

## 1. Introduction

The brachialis muscle is the major elbow flexor. The brachialis tendon inserts distally to the coronoid process at the tuberosity of the ulna. This distal attachment has been described by previous studies as fibers of the brachialis muscle converging to a thick, broad tendon which is attached to the tuberosity of the ulna and to a rough impression on the anterior aspect of the coronoid process [1–3]. It is innervated by branches of the musculocutaneous and radial nerves [4, 5].

Injury to the brachialis is uncommon and reports are often limited to small case series or case reports [6, 7]. As a result, there has been little anatomical characterization of the distal attachment of the brachialis muscle [1]. According to Cage et al. [8], the brachialis muscle is injured during type III coronoid fractures that involve disruption of more than 50% of the height of the coronoid process, affecting the brachialis distal tendinous insertion at the more proximal aspect of the ulnar tuberosity.

Previous research on the muscle footprints took measurements with calipers and MRI as illustrated in Table 1. Three-dimensional characterization allows for a more objective morphometric analysis of the muscle footprint. This study provides information on the qualitative and quantitative morphometry of the distal brachialis muscle insertion on the ulna. The knowledge obtained from the study contributes to our understanding of the functional and anatomic characteristics of the brachialis muscle.

A better understanding of the muscle footprint could be used to improve the current anterior and anterolateral surgical approaches to repair fractures around the elbow joint with ulnar bony involvement [1]. Although injury to the brachialis muscle or tendon is rare, information from this study could serve as a guide for orthopedic surgeons considering the use of the brachialis tendon as a treatment option in cases like brachialis tendon injuries, biceps brachii tendon ruptures, brachial plexus injury, and coronoid process fractures [2, 9].

## 2. Materials and Methods

Fifty cadaveric upper limbs from twenty-eight formalin preserved cadavers (University of Kentucky) were dissected from the proximal segment of the humerus distally to the wrist, leaving the distal insertions of the brachialis intact. The muscle was stripped off the distal humerus and scissor dissection was used to free the brachialis from its surrounding soft tissue attachments on the anterior capsule and in the antecubital region. The limbs of twenty-one cadavers were dissected bilaterally, while eight were dissected unilaterally. Next, each dissected elbow was secured in a customized jig and the data was acquired by tracing the footprints of the brachialis insertion and the proximal ulna using a three-dimensional digitizer (FARO, Faro Technologies, Lake Mary, FL, USA) mounted with a 2 mm ball probe (Renishaw, Gloucestershire, UK).

**Figure 1:**
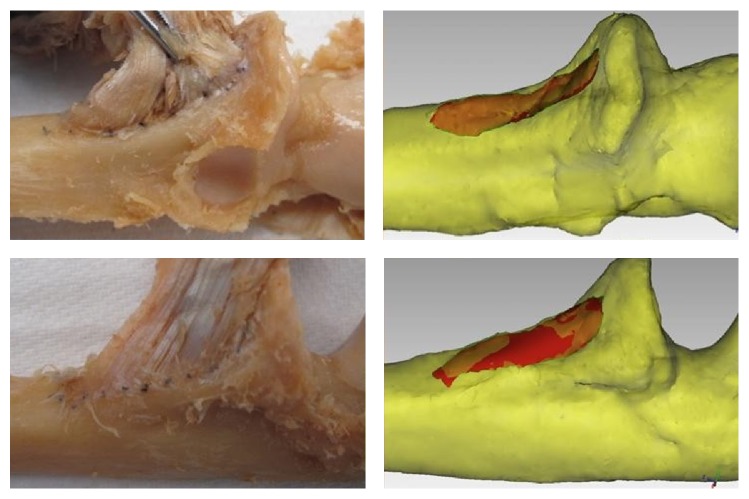
The cadaveric brachialis on the ventral surface of the left arm inserting into the coronoid process of the ulna and the tuberosity of the ulna and its digitized counterpart.

The digitized specimens were registered on a three-dimensional inspection computer software, Geomagic Qualify version 12 (Geomagic, Research Triangle Park, NC). The established muscle footprints on the intact elbow bones produced a model to obtain accurate measurements of the muscles with respect to the length, width, and area at their distal insertions. The muscles were then characterized based on their shape and relative locations (Figure 1).

**Figure 2:**
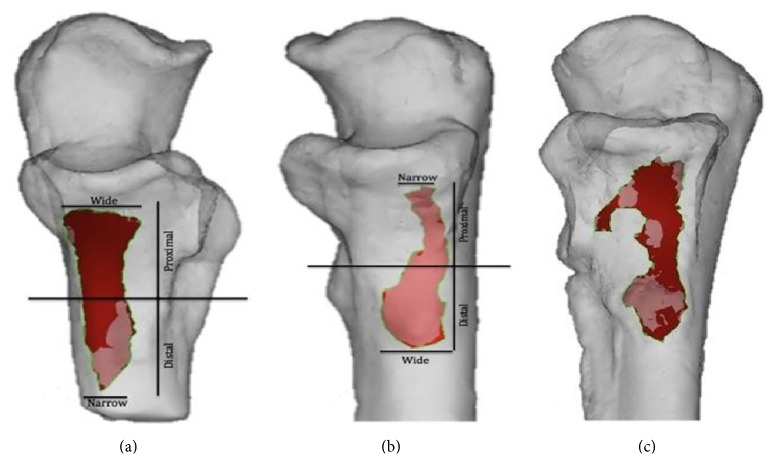
Illustration of brachialis insertion onto ventral surface of left coronoid and tuberosity of ulna shape analysis. (a) Proximal wide and distal narrow shape (*n* = 30). (b) Proximal narrow and distal wide shape (*n* = 15). (c) Miscellaneous (*n* = 5).

**Figure 3:**
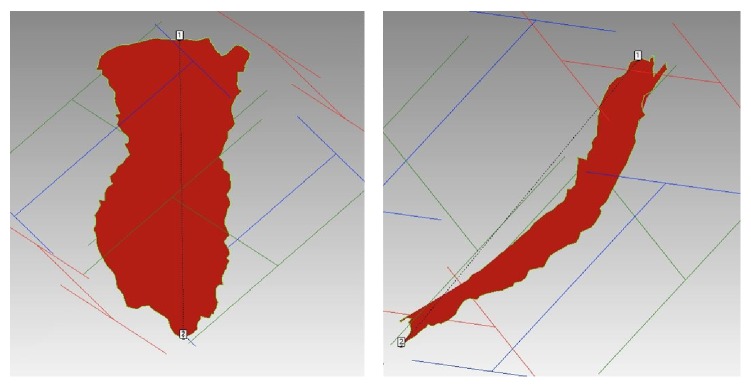
Measurement of brachialis projected and surface length and width and area.

Due to the irregularity and variability of the shapes [11], they were classified as being proximal or distal in relation to the widest point of the footprint; top and bottom halves of the footprints were described as narrow or wide relative to each other as illustrated in Figure 2. The length and width of the footprint projection were measured as lines connecting the distal most points of the projection lengthwise and widthwise; the second measurement, the surface length and width, were taken using the same points on the bone surface with the contours of the bone taken into consideration (Figure 3). The width measured was the maximum width of the footprint anywhere along its length. The area of the muscle insertion was derived directly using the computer software.

A one-way ANOVA test was used to determine the presence of any statistical difference between males and females and between the left and right sides. The statistics package SPSS version 20 was used (SPSS, IBM corporations, Somers, NY). A *P* value of less than 0.05 was considered significant.

The data obtained for this study were divided into two components. The first component entailed the qualitative analysis of the shape of all 50 specimens. The second component of the study involved a quantitative description of the length, width, and area of the muscle insertion on 23 specimens. All of the data presented here was acquired using a ball probe.

## 3. Results

**Figure 4:**
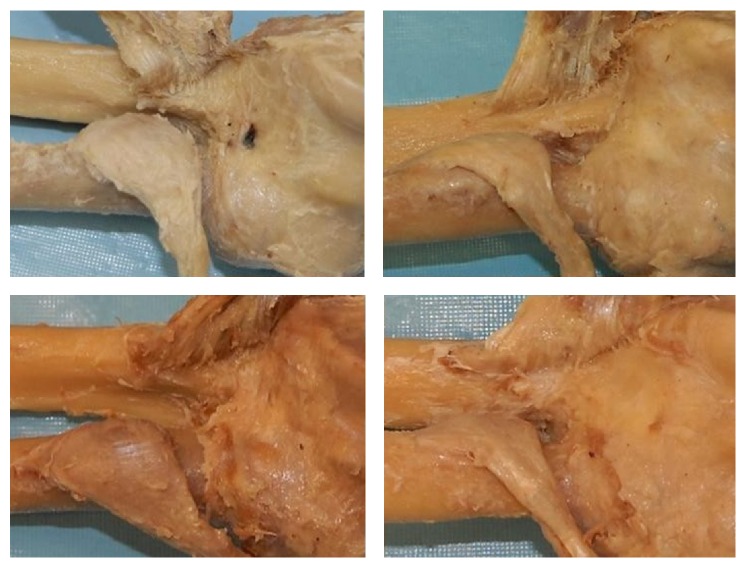
Examples of brachialis footprint shape variations at its insertion point on the tuberosity of coronoid of the ulna.

The qualitative results combined the first study of 27 cadaveric elbows and the second study of 23 cadaveric elbows. Fifty brachialis footprints were described in total: 27 of those footprints were located on the right side and 23 were located on the left side. The mean donor age was 81.3 years (range: 68–92 years). Due to the shape variation of the 50 brachialis footprints (Figure 3), the shapes of 30 were described as proximally narrow and distally wide, 15 were described as proximally wide and distally narrow, and 5 footprints were irregularly shaped and classified as miscellaneous (Figure 4).

The measurements of the brachialis footprint were based on the data collected from 23 elbows, 12 on the right side and 11 on the left side (Table 2). When considering the length of the footprints, the measurements were divided based on projected length and surface length (with the contours of the ulnar bone taken into consideration) (Figure 3). The mean projected length of the footprint was 32.2 ± 3.6 mm (range, 22.2–38.2 mm), mean surface length was 33.3 ± 3.7 mm (range, 22.6–40.1 mm), mean projected width was 9.3 ± 2.3 mm (range, 4.9–13.2 mm), mean surface width was 9.6 ± 2.3 mm (range, 5.3–13.6 mm), and mean surface area was 224.5 ± 67.1 mm (range, 128.3–398.3 mm).

When the results were compared by gender, only the surface length was found to be significantly larger in males compared to females, *P* = 0.002 (Table 3). When the results were compared by side, no significant difference existed between any of the measurements (Table 4).

## 4. Discussion

We set out to describe the three-dimensional morphometry of the brachialis muscle at its distal attachment on the ulna. The results from this study indicate that the projected and surface lengths of the brachialis footprint are significantly longer in males compared to females. The projected and surface width and the surface area, however, were not found to be significantly different between males and females. The lengths, widths, and surface area of the left distal footprint were consistently larger than the footprints on the right side, but these results were not statistically significant. The shapes were described as proximal narrow and distal wide, proximally wide and distal narrow, or miscellaneous, which may represent true geometric variation or different stages of age related degenerative change.

According to Cage et al. [8], Leonello et al. [1], and Sanal et al. [2], the insertion has been described as a thick, broad tendon or as having two heads, one with a tendinous and the other with an aponeurotic attachment. The superficial head is more distal to the ulnar tuberosity and is in the form of a thick tendinous structure, while the deep head is more proximal to the ulnar tuberosity and is musculoaponeurotic, fan shaped, and broad at its site of attachment. Although they differ at their distal ends, the two heads attach to the ulnar tuberosity as a single blended structure, which was consistent with our observations. This allows the brachialis muscle to be the primary flexor of the elbow joint regardless of whether the forearm is in pronation or supination [12]. Most of the brachialis insertion was medial to the line of olecranon-coronoid tip, which was consistent with results from Ma and Chang [10].

**Figure 5:**
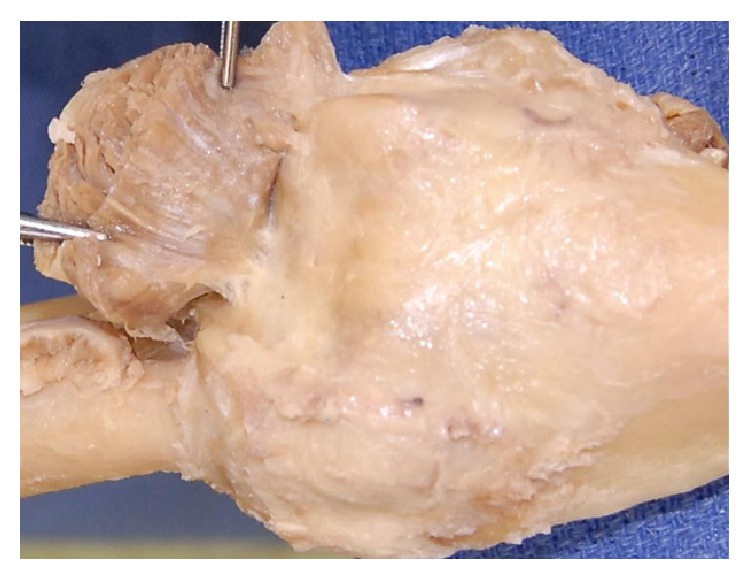
Attachment of the brachialis muscle fibers to the anterior capsule.

Some reports indicated that fibers of the brachialis muscle had no direct attachment to the anterior capsule of the elbow joint [13] but rather a connection between the muscle and anterior capsule that existed via connective tissue [2, 10, 11]. According to Leonello et al. and Morrey [1, 14], a small collection of fibers, articularis cubitus, extend from the deep aspect of the deep head of the brachialis muscle and attach directly to the anterior capsule of the elbow joint. Other reports found both a combination of muscle fibers and connective tissue that connected the brachialis muscle to the anterior elbow capsule [15]. Deep aspect brachialis muscle fibers were observed to attach directly to the capsule in 19 of our specimens (Figure 5).

When the brachialis footprints were compared among different studies (Table 1), the measurements from our study were in between the range from all the studies listed. These variations could be attributed to several reasons such as the differences in patient demographics, data measurement techniques, data acquisition and analysis, and age and number of specimens utilized in each study.

With regard to the limitations of the study, firstly, the anatomic study was conducted with cadaveric specimens derived from elderly donors, without a younger age spectrum for comparison. Secondly, no histological analysis was carried out to determine the nature of the insertion of the muscle on the ulnar tuberosity. This made our qualitative analysis purely observational. Therefore, the presence of tears (due to dissection) and removal (during the disarticulation of the elbow joint) of the muscle fibers that connected to the elbow capsule [2] might have contributed to some sampling errors during data collection and analysis. Thirdly, the total number of samples was relatively small, potentially inadequate to achieve high statistical power. Finally, not all of the footprints were quantitatively analyzed because of mismatch errors that occurred when using the laser scanner and ball probe in combination. Hence quantitative analysis was confined to those specimens with matching laser and ball probe data.

## 5. Conclusion

This study provides information on the qualitative and quantitative morphometry of the distal brachialis muscle insertion on the ulna, in an older population. The knowledge of the anatomic description of the distal insertion of the brachialis muscle aids in comprehending the functional and anatomic characteristics of the muscle, without automatically inferring the existence of pathological changes when the morphology has age related alterations. This information would not only greatly enhance the understanding of the natural history and kinematics of the brachialis but could be used to vastly improve the current anterior and anterolateral surgical approaches used to repair fractures around the elbow joint.

## Figures and Tables

**Table 1:** Comparison of the brachialis distal footprint dimensions among different studies.

Study	Brachialis length (mm)	Brachialis width (mm)	Footprint area (mm^2^)	Number of specimens and gender/side	Mean age (years)	Data acquisition & analysis
Cage et al. [8]	26.3 (range 17.6–33.9)	10.3 (range 3.3–21.3), proximally 4 (range 1.5–6.9), distally	N/A	20: 10 males, 8 females, 2 unknown 20: 8 right, 12 left	76	Digital calipers and computer software

Leonello et al. [1]	44 (range 28–53)	N/A	N/A	11: not specified	N/A	N/A

Ma and Chang [10]	21.79 ± 2.70	4.11 ± 1.12	N/A	8: not specified	67.8	Vernier calipers

Current study	33.3 ± 3.7	9.6 ± 2.3	224.5 ± 67.1	23: 11 males, 12 females	81.3	3-dimensional digitizer and computer software

N/A = not available.

**Table 2:** Brachialis insertion measurements and shape analysis from twenty-three cadaveric elbows.

Cadaver number	Sex	Side	Projected length (mm)	Surface length (mm)	Projected width (mm)	Surface width (mm)	Surface area (mm^2^)
1	Female	Right	30.2	32.0	8.7	8.8	167.4
		Left	28.0	29.6	7.3	7.3	155.0
2	Male	Right	31.0	31.6	7.6	7.7	171.8
		Left	36.0	36.6	8.5	8.8	255.4
3	Female	Right	32.6	33.7	10.1	10.6	224.5
		Left	28.9	29.9	8.2	8.3	157.2
4	Male	Right	35.9	36.6	6.2	6.3	193.0
		Left	33.4	35.3	4.9	5.3	133.9
5	Female	Right	29.4	30.9	13.0	13.6	275.9
		Left	29.7	30.8	12.7	12.7	234.6
6	Male	Right	31.3	32.6	11.0	11.2	302.7
7	Female	Right	22.2	22.6	9.3	9.8	147.3
		Left	30.4	31.6	7.8	7.9	182.2
8	Female	Right	31.0	31.5	10.1	10.4	242.6
		Left	30.8	32.3	7.6	7.7	205.2
9	Male	Right	35.8	37.0	13.2	13.5	398.3
		Left	38.2	40.1	12.9	12.9	323.9
10	Male	Right	31.5	32.5	9.1	9.1	242.7
		Left	32.9	33.7	7.2	7.2	128.3
11	Female	Right	33.3	35.7	12.4	12.7	261.1
		Left	33.5	34.2	8.5	8.9	213.1
12	Male	Right	36.0	36.9	9.7	10.0	283.2
		Left	38.1	39.1	9.0	9.6	265.3
Mean			32.2	33.3	9.3	9.6	224.5
SD			3.6	3.7	2.3	2.3	67.1

**Table 3:** Comparison of the brachialis dimensions by gender.

Number of specimens	Mean Projected length ± SD (mm)	*P* value	Mean surface length ± SD (mm)	*P* value	Mean projected width ± SD (mm)	*P* value	Mean surface width ± SD (mm)	*P* value	Mean area ± SD (mm^2^)	*P* value
Males (*n* = 11)	34.6 ± 2.6	0.001	35.6 ± 2.8	0.002	9.0 ± 2.6	0.534	9.2 ± 2.6	0.514	245.3 ± 83.1	0.160
Females (*n* = 12)	30.0 ± 3.0		31.2 ± 3.2		9.6 ± 2.0		9.9 ± 2.1		205.5 ± 43.6	

SD = Standard Deviation; *n* = number of specimens.

**Table 4:** Comparison of the brachialis dimensions by side.

Number of specimens	Mean projected length ± SD (mm)	*P* value	Mean surface length ± SD (mm)	*P* value	Mean projected width ± SD (mm)	*P* value	Mean surface width ± SD (mm)	*P* value	Mean area ± SD (mm^2^)	*P* value
Right (*n* = 12)	31.7 ± 3.8	0.505	32.8 ± 3.9	0.480	10.0 ± 2.1	0.138	10.3 ± 2.2	0.119	242.5 ± 69.7	0.185
Left (*n* = 11)	32.7 ± 3.5		33.9 ± 3.6		8.6 ± 2.3		8.8 ± 2.3		204.9 ± 61.2	

SD = Standard Deviation; *n* = number of specimens.
